# Temperature-Related Reaction Norms of Gene Expression: Regulatory Architecture and Functional Implications

**DOI:** 10.1093/molbev/msv120

**Published:** 2015-05-14

**Authors:** Jun Chen, Viola Nolte, Christian Schlötterer

**Affiliations:** ^1^Institut für Populationsgenetik, Vetmeduni Vienna, Wien, Austria/Europe

**Keywords:** transcriptome, expression plasticity, mRNA-seq, reaction norm, gene expression regulation

## Abstract

The environment has profound effects on the expression of many traits and reaction norms describe the expression dynamics of a trait across a broad range of environmental conditions. Here, we analyze gene expression in *Drosophila melanogaster* across four different developmental temperatures (13–29 °C). Gene expression is highly plastic with 83.3% of the genes being differentially expressed. We distinguished three components of plasticity: 1) Dynamics of gene expression intensity (sum of change), 2) direction of change, and 3) curvature of the reaction norm (linear vs. quadratic). Studying their regulatory architecture we found that all three plasticity components were most strongly affected by the number of different transcription factors (TFs) binding to the target gene. More TFs were found in genes with less expression changes across temperatures. Although the effect of microRNAs was weaker, we consistently noted a trend in the opposite direction. The most plastic genes were regulated by fewer TFs and more microRNAs than less plastic genes. Different patterns of plasticity were also reflected by their functional characterization based on gene ontology. Our results suggest that reaction norms provide an important key to understand the functional requirements of natural populations exposed to variable environmental conditions.

## Introduction

Plasticity is the phenomenon of a single genotype producing different phenotypes in response to environmental changes. It has been observed for many phenotypic traits across a broad range of organisms (Pigliucci 2001; Whitman and Ananthakrishnan 2009). Phenotypic plasticity may result from physiological or behavioral interactions with the environment (Ghalambor et al. 2007). One possible outcome of phenotypic plasticity is a change in fitness, which could affect the frequency of phenotypes in the population in the absence of genetic changes (Carroll et al. 2007; Rasanen and Kruuk 2007). Thus, it can be a powerful and effective mechanism to buffer detrimental effects of short-term environmental changes (e.g., Fischer and Karl 2010). Recent studies show that plasticity can be adaptive and even speed up genetic adaption when combined with natural selection (Price et al. 2003; West-Eberhard 2003; Ghalambor et al. 2007). On the other hand, a plastic response can either prevent adaptation (Falconer 1981) or even be deleterious if it shifts the phenotype away from the adaptive peak.

The capacity of a given genotype to modulate its phenotype is often described by the reaction norm, which can be modeled by a mathematic function describing how the phenotype changes across a range of environmental conditions. A well-known example is the temperature-size law, which was first introduced by Atkinson (1994) and describes the negative correlation of developmental temperature and the body size in ectotherms. Reaction norms have been found to differ quite widely among traits (e.g., David et al. 1997; Whitman and Ananthakrishnan 2009; Klepsatel et al. 2013). Although the shape of the reaction norm could potentially provide information about the underlying regulatory architecture of a given trait, we are still lacking an interpretative framework. Thus, reaction norms currently serve primarily as a descriptive variable with no predictive or explanatory power.

This situation may change when more traits are being studied for the same set of environmental conditions such that sufficient power is provided to link the shape of reaction norms to their underlying regulatory architecture. As expression can be estimated for a large number of genes in a single experiment, it provides an excellent opportunity to study the reaction norms for many traits and identify common patterns of genes with similar reaction norms. Despite being not yet well investigated, an increasing number of studies are reporting gene expression under multiple environmental conditions in yeast, *Drosophila*, *Daphnia*, and others (e.g., Causton et al. 2001; Levine et al. 2011; Telonis-Scott et al. 2013; Yampolsky et al. 2014). Although it is apparent that many genes have plastic gene expression, so far only limited efforts have been made to model gene expression changes along an environmental gradient or the underlying regulatory architecture. Most likely, this gap is partially caused by the focus on the response to extremely stressful conditions such as cold/heat shock, or presence/absence of chemicals (reviewed in Lopez-Maury et al. 2008).

Temperature is a major environmental factor, in particular for ectotherms, such as *Drosophila*. To account for the impact of temperature, ectotherms invoke a broad range of physiological and behavioral responses (Gibert and De Jong 2001). The comparison of the phenotypes of genetically identical individuals grown at different temperatures is frequently used to determine the reaction norms of various traits, including body size, growth rate, lifespan, and fecundity in *Drosophila* (e.g., Economos and Lints 1986; David et al. 2011; Klepsatel et al. 2013). Although the shape of the reaction norm differs among the traits studied, the large influence of temperature is apparent.

In this study, we measure gene expression using RNA-Seq in *D. melanogaster* females across a broad temperature range and classify the reaction norm of each gene. Based on the pattern of expression plasticity we identify groups of genes with a common regulatory architecture and similar functional categories, as defined by gene ontology (GO) analyses.

## Results

**Fig. 1.**
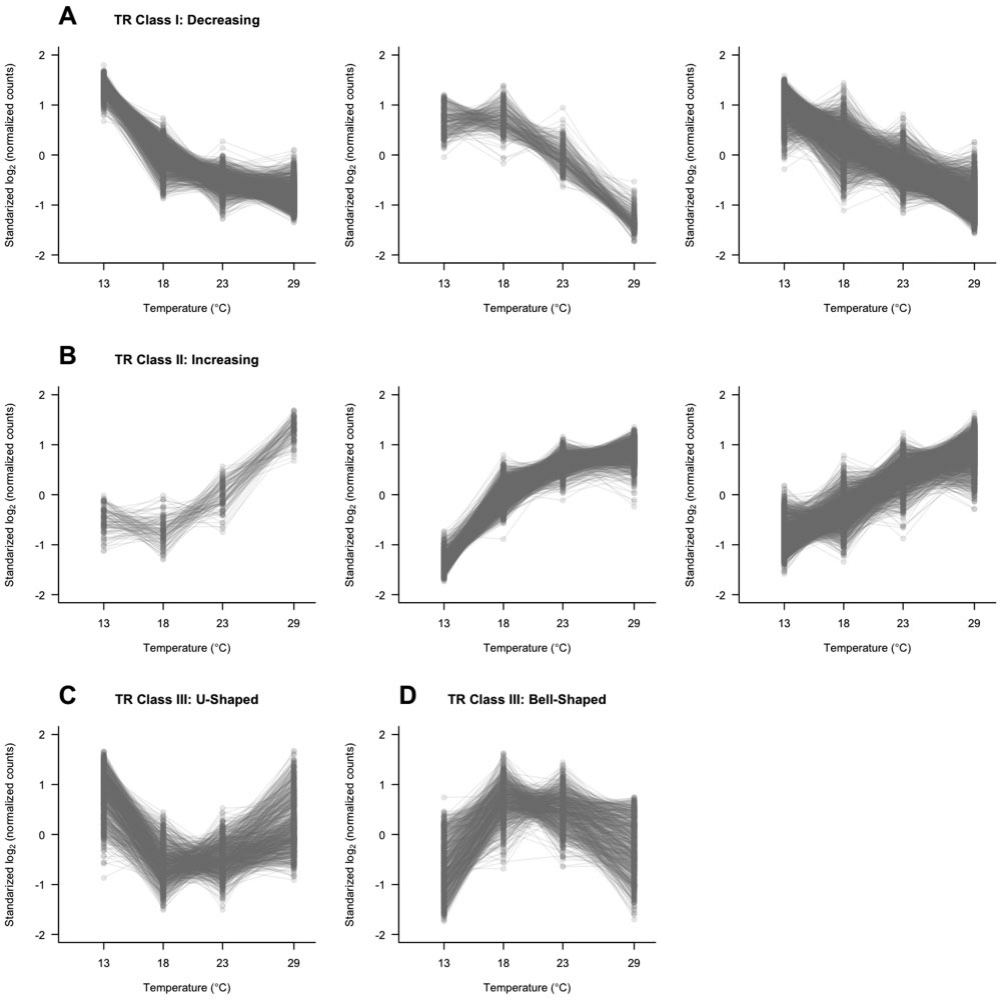
Classification of genes based on the expression plasticity. Read counts were normalized and then standardized to have a mean value equal to zero and variance equal to 1 (for illustration only). Median values for all three replicates in each temperature are presented as solid dots for each gene. (*A*) Class I: Genes with expression levels decreasing with temperature. (*B*) Class II: Genes with expression levels increasing with temperature. For (*A*) and (*B*), panels from left to right present the genes fitted in positive quadratic, negative quadratic, and linear model, respectively. (*C*, *D*) Class III/IV: Genes with min/max expression levels at the temperature between 18 and 23 °C.

Gene expression was measured by RNA-Seq in F1 individuals from a cross of two inbred lines, Oregon R and Samarkand, which developed at four temperatures (13, 18, 23, and 29 °C), each in three replicates. On average, about 27 million of 43 million read pairs (63%) could be mapped to annotated gene features in the reference genome and counted unambiguously, with higher percentages found in 23 and 29 °C (see supplementary table S1, Supplementary Material online). To account for the differences in read depth and expression differences between libraries, we performed trimmed mean of *M*-values method (TMM) normalization (see Materials and Methods) and only focused on genes expressed in all 24 libraries. In total 9,995 of 18,764 *D. melanogaster* genes (53%) were expressed at all temperatures, which corresponds to 70% of the protein-coding genes annotated in Flybase. 83.3% of the genes expressed at all temperatures were plastic, showing differential expression among the temperatures. We further characterized the plasticity of gene expression using three different components of plasticity: 1) Dynamics of gene expression intensity, measured by the sum of change (SOC, see Materials and Methods); 2) curvature: We distinguished between linear and quadratic curvatures (Gibert et al. 1998); and 3) direction of change. In total, 3,295 genes (33%) had a linear curvature and 5,033 genes (50.4%) followed a quadratic reaction norm (fig. 1 and table 1). We distinguished four directions of gene expression changes: In 3,350 genes (33.5%), the expression intensity increased with temperature and for 3,430 genes (34.3%) it decreased. In addition, a moderately sized group of genes did not show a monotonous change in gene expression, which was classified into U-shaped (818 genes, 8.2%) and bell-shaped (730 genes, 7.3%) (fig. 1 and table 1; see Materials and Methods for details of each class). In total, 1,667 genes (16.7%) did not show significant expression changes or the fold changes were smaller than 1.25 across temperatures. We classified them as a group of genes with conserved expression. A detailed list can be found in supplementary data set S1, Supplementary Material online, that contains information about reaction norm model and expression classification for each gene. Temperature-specific gene expression was found to be rare: Only 424 genes are expressed at one temperature, 255 at two temperatures, and 215 at three temperatures. We focused on 9,995 genes expressed at all temperatures in our analyses to identify general regulatory features of temperature-dependent gene expression.

**Table 1.** The Classification of Gene Expression Reaction Norm.

Conserved	Increasing		Decreasing		U-Shaped	Bell-Shaped
	l.m.	quad.	l.m.	quad.		
1,667	1,403	1,947	1,892	1,538	818	730

### Linking Plasticity with Regulatory Architecture

Reasoning that gene expression plasticity results from differential gene regulation, we identified five factors that could capture some of the complexity of the underlying regulatory architecture. As transcription factors (TFs) and microRNAs are important factors shaping gene expression, we used the number of different TFs/microRNAs binding to each gene to estimate the *trans*-regulatory contribution. In addition, we used the length of the 5′- and 3′-untranslated regions (UTRs). The 3′-UTR length is correlated with the number of microRNA-binding sites (Stark et al. 2005). As the first intron is frequently involved in gene regulation (Gaffney and Keightley 2006), we also expected a positive correlation between intron length and the number of regulatory targets, as we did for the UTRs. The influence of these five factors was tested on each of the three plasticity components—SOC, direction of change, and curvature. In total, we analyzed 5,138 genes that harbor all five factors including 2,516 linear genes and 2,622 quadratic genes (U- and bell-shaped genes excluded). In total, 2,468 genes showed increasing expression intensities with temperature whereas for 2,670 genes expression intensity decreased with increasing temperature.

#### Sum of Change

We used generalized linear models (GLMs) to determine how the five components of the regulatory landscape affected the dynamics of gene expression intensity (SOC). According to the “Akaike Information Criterion” (AIC), the model was sufficiently explained by the number of TFs and microRNAs and their interaction (table 2). Genes with more TFs had less dramatic expression changes (lower SOC values, *P* value < 2e-16). The opposite effect was seen for microRNAs, where a larger number of microRNAs were associated with stronger gene expression differences (*P* value < 6.7e-8). The number of TFs had a stronger effect on SOC (coef. = −3e-3) compared with microRNAs (coef. = 1.8e3). As expected, the number of TFs explained much more of the total variance (adjusted *R*^2 ^= 6.7%) than microRNAs (0.4%) and their interaction (0.16%).

**Table 2.** Summary of GLMs for Regression of SOC on the Number of TFs and miRNAs.

	Coefficient	*P* Value	*R*^2^ (%)^a^
SOC∼glm (TFs + microRNA + TFs: microRNAs)			7.3
(Intercept)	1.125	<2e-16	
TFs	−3e-3	<2e-16	6.76
microRNAs	1.8e-3	6.65e-08	0.4
TFs: microRNAs	−6.2e-5	1.5e-3	0.165

^a^Pseudo-*R*^2^ calculated as explained deviance for the full model and for each variable. *R*^2^ for SOC, curvature, and direction were calculated by comparing *R*^2^ between the full model and a reduced model with the component and its interactions excluded.

#### Direction of Change

We performed the linear discriminant analysis on five components of the regulatory architecture. We randomly picked 60% of the genes in the total data set as training set to build the model. The most pronounced effect (coef. = 0.091) was seen for the number of TFs: Genes with increasing gene expression intensity (class I) were regulated by more TFs than those with a decreasing gene expression intensity (class II). The opposite trend, albeit less strong (coef: = −0.03), was seen for microRNAs. The influence of the other factors was weak (table 3). In order to evaluate how accurately the model explains the direction of change, we applied it to the remaining 40% of the loci and classified 75% of them correctly. An even higher accuracy (80%) was obtained with the nonparametric random forest model. Interestingly, all five regulatory components were important for the accuracy of the prediction (supplementary fig. S1, Supplementary Material online).

**Table 3.** Summary of Discriminant Analyses on Direction of Change and Curvature Based on Five Components of Regulatory Architecture.

	TFs^a^	MicroRNAs^a^	5′-UTR^b^	3′-UTR^b^	First Intron^b^
Direction					
Decrease	11.7	11.6	373.2	868	4,078.2
Increase	18.2	7.1	208.8	471.8	838.8
Coefficient	0.091	−0.03	−1.5e-3	−1.3e-4	−4.9e-5
Accuracy	0.753^c^ (0.798^d^)				
Curvature					
Linear	14.0	10.2	305.2	735.5	2,638.2
Quadratic	15.7	8.8	284.5	624.2	2,427.7
Coefficient	0.082	−0.05	−5.5e-4	−3e4	1.8e-5
Accuracy	0.542^c^ (0.516^d^)				

^a^Mean number is shown.

^b^Mean length is shown.

^c^Predicted using LDA.

^d^Predicted using random forest analysis.

#### Curvature

We applied the same discriminant analyses for classification of curvature and obtained similar results as for the direction of change. The number of TFs had the most pronounced effect (coef. = 0.082), with genes with a quadratic reaction norm being regulated by more TFs than genes with a linear reaction norm. MicroRNAs had a weaker effect, but in the opposite direction. Genes with a quadratic reaction norm were regulated by fewer microRNAs. However, the accuracy of prediction on curvature classification was low (0.54) and could not be distinguished from the class proportion of the training set (0.49 and 0.51 for linear and quadratic curvatures; table 3). This might suggest that compared with the direction of change the classification of curvature in this study is more difficult and more expression data are needed at a finer resolution of the temperature range. However, it could also mean that the underlying regulatory architecture is more complicated and our analysis is not sufficient to describe the difference in regulation between genes falling into the two curvature classifications.

### Linking Plasticity with Functional Requirements

To test whether the shape of the reaction norms reflects a change in the functional requirements of *D. melanogaster* across different developmental temperatures, we applied a GO enrichment analysis. Regardless of direction, no significant enrichment of any functional class could be identified in the comparison of genes with linear and quadratic reaction norms. We neither found a strong enrichment among genes with similar gene expression intensity across the surveyed temperature range (see supplementary data set S2, Supplementary Material online). In contrast, we observed many and highly significant enrichments of GO categories for genes with increasing and decreasing reaction norms as well as bell-shaped and U-shaped ones.

#### Class I: Genes with Increasing Expression Level in the Cold Are Enriched for Ion Transport and Signaling

For 3,430 genes, the expression intensity decreased from 13 to 29 °C. Out of these 520 (15%) were enriched in 50 hierarchical GO categories related to transmembrane activities, including voltage-gated/ligand-gated channels, and transmembrane transport. In total, 358 (10%) genes were enriched in 14 GO categories with signaling pathways and neurotransmitters. In addition to ion transport and signaling, also some morphological terms, such as genital disk development and cell fate specification showed a high enrichment, but due to the small number of genes involved the enrichment was only moderately significant (see supplementary data set S2, Supplementary Material online).

One possible explanation for the generally higher expression levels of ion transporters may be related to the temperature-dependent activity of ion channels. Generally speaking ion channel conductance seems to be lower at low temperatures (Hoffmann and Dionne 1983; Klockner et al. 1990; Milburn et al. 1995), thus a higher expression may compensate for the lower signaling activity to generate signaling homeostasis.

#### Class II: Genes with Increasing Expression Level in the Hot Are Enriched for Metabolic Processes and Cell Cycle Genes

The expression level of 3,350 genes increased from 13 to 29 °C. Among these genes we detected a very strong enrichment for genes involved in metabolic processes and cell cycle. In total, 1,314 genes (39%) were overrepresented in 50 GO categories related to macromolecule metabolism, including DNA, RNA, and protein metabolism (see supplementary data set S2, Supplementary Material online). Consistent with an increasing transcription level at higher temperatures, we also noted the enrichment of genes involved in chromatin organization and histone modification. Among them were several genes associated with H3K4 methylation, a mark correlated with active transcription.

In addition to the increased metabolic activity at higher temperature, we found that 1,853 genes were enriched in 80 GO categories functionally related to cell division and cell cycle regulation (see supplementary data set S2, Supplementary Material online). As we also observed a significant enrichment of genes involved in DNA repair, we conclude that DNA damage might increase with higher temperatures and that the cell cycle control genes may be involved in delaying the cell cycle to provide time for damage repair (Maldonadocodina et al. 1993; Elledge 1996; Sekelsky et al. 1998).

#### Class III: Genes with the Highest Expression Level at Intermediate Temperatures Are Enriched in Reproduction-Related Genes

The reaction norm of 730 genes was found to be bell-shaped. As we assumed that the difference between maximum and minimum expression is an important factor, we used the quadratic term to rank these 730 genes. Chorion genes showed the most dramatic change in gene expression across the entire temperature range, suggesting that egg production was highest at 18 and 23 °C, but strongly reduced at more extreme, stressful temperatures. This interpretation is backed not only by our observation that egg laying was dramatically reduced at these temperatures (data not shown) but also by other studies measuring reaction norms for life-history traits (Delpuech et al. 1995; Wayne and Mackay 1998; Klepsatel et al. 2013).

#### Class IV: Genes with Lowest Expression at Intermediate Temperature Are Enriched in Immune-Related Genes

In total, 818 genes had a U-shaped reaction norm. GO analysis based on ranked quadratic coefficients revealed an enrichment of defense response genes, mainly involved in antibacterial humoral response (see supplementary data set S2, Supplementary Material online). Consistent with our results, previous studies found that exposure to cold can lead to upregulation of several immune-related genes in *D. melanogaster* including AttA, AttB, and IM23 (Zhang et al. 2011). Furthermore, cold temperature also resulted in a higher resistance to fungal infection (Le Bourg et al. 2009). A comparison of three populations at 18, 23, and 28 °C showed a significantly lower bacterial load at 28 °C (Lazzaro et al. 2008). A study on honeycomb larvae also showed an increased immune response after heat-shock (Wojda and Taszlow 2013). Interestingly, the complementary expression pattern observed for reproduction and immunity-related genes may indicate a tradeoff between these two traits. Such tradeoff has been previously suggested to be caused by reallocation of energetic resources between immunity and other components of fitness including egg production and hatch rate (Carton and David 1983; Fellowes et al. 1999; Kraaijeveld et al. 2002; Luong and Polak 2007; Rashed et al. 2008).

### Expression of Hsp Genes

One of the best-studied functional consequences of temperature stress is the expression of heat shock proteins (Parsell and Lindquist 1993). Contrary to our study, the expression of Hsp genes is typically studied in the context of rapid exposure to extreme environmental temperatures or other stressors. In spite of a strong dependence on genotypes and assaying temperatures, some Hsp genes have been frequently shown to be involved in the response to temperature stress, with the *Hsp70* family and *Hsp90* being the most prominent genes (Parsell and Lindquist 1993). Given the obvious link between temperature and Hsp expression, we analyzed 19 Hsp genes for which the gene expression pattern could be determined in our data set (table 4).

**Table 4.** Expression Plasticity of Hsp Genes at Different Development Temperatures.

Family	Gene Name	Flybase ID	Plasticity
*HSP90*	*Hsp83*	FBgn0001233	Conserved
*HSF*	*Hsf*	FBgn0001222	Increasing
*DnaJ*	*Hsc20*	FBgn0263606	Increasing
	*Hsp40*	FBgn0263106	Increasing
*HSP60*	*Hsp60*	FBgn0015245	Increasing
	*Hsp60B*	FBgn0011244	n.e.
	*Hsp60C*	FBgn0031728	n.e.
	*Hsp60D*	FBgn0032525	n.e.
Small heat shock protein (*HSP20*)	*Hsp22*	FBgn0001223	n.e.
	*Hsp23*	FBgn0001224	U-shape
	*Hsp26*	FBgn0001225	Increasing
	*Hsp27*	FBgn0001226	Increasing
	*Hsp67Ba*	FBgn0001227	Decreasing
	*Hsp67Bb*	FBgn0001228	n.e.
	*Hsp67Bc*	FBgn0001229	Decreasing
*HSP70*	*Hsp68*	FBgn0001230	Decreasing
	*Hsp70Bb*	FBgn0013278	Decreasing
	*Hsp70Bc*	FBgn0013279	Decreasing
	*Hsc70-1*	FBgn0001216	Decreasing
	*Hsc70-2*	FBgn0001217	Decreasing
	*Hsc70-3*	FBgn0001218	Conserved
	*Hsc70-4*	FBgn0266599	n.a.
	*Hsc70-5*	FBgn0001220	U-shaped
	*Hsc70-6*	FBgn0001221	n.a.
	*Hsc70Cb*	FBgn0026418	U-shaped
	*Hsp70Aa*	FBgn0013275	n.e.
	*Hsp70Ab*	FBgn0013276	n.e.
	*Hsp70Ba*	FBgn0013277	n.e.
	*Hsp70Bbb*	FBgn0051354	n.e.
/	*HSE*	FBgn0041631	n.e.
/	*Hsp64*	FBgn0020649	n.a.
Non-protein coding	*Hsrω*	FBgn0001234	Decreasing

Note.—n.e., not expressed; n.a., not annotated.

*Hsp83* (also known as *Hsp90*) together with *Hsc70-3* are the only Hsps that were not affected by different developmental temperatures used in this study. The rather constant gene expression of *Hsp90* could be explained by the fact that the range of developmental temperatures applied in our experiment was not stressful enough to induce an upregulation of *Hsp90*. While after a short-term exposure at 31 °C, 2 °C more than the highest temperature used in our experiment, Colinet et al. (2013) have shown that *Hsp90* can be significantly upregulated. On the other hand, six Hsp genes (*Hsf*, *Hsc20*, *Hsp40*, *Hsp26*, *Hsp27*, and *Hsp60*) showed an increase of expression with the temperature and eight Hsp genes (*Hsp67Ba*, *Hsp67Bc*, *Hsp68*, *Hsp70Bb*, *Hsp70Bc*, *Hsc70-1*, *Hsc70-2*, and *Hsrω*) had decreased expression levels at higher temperatures. The observation of temperature-dependent expression change in those Hsp genes may suggest that even without extreme temperature shocks they still serve an important function for temperature adaptation. However, the direction of expression changes is not always conclusive for every Hsp family when compared with heat shock experiments. Although *Hsp60* is upregulated after heat shock and at higher temperatures in our experiment, we find that members of the *Hsp70* family are downregulated in the hotter environments, whereas other studies reported upregulation after heat shock.

## Discussion

Our study demonstrated a high level of plasticity in gene expression across different temperatures—up to 83% of the expressed genes differed in their gene expression level over four different developmental temperatures. This observation contrasts previous studies in *D. melanogaster*, which only found moderate differences in gene expression between flies developed at different temperatures (Levine et al. 2011; Zhou et al. 2012). We attribute this difference to the combined effect of the higher power for RNA-Seq relative to microarrays used in the previous reports and a larger number of temperatures surveyed in this study.

### Regulatory Architecture

One novel aspect of our study is the analysis of the regulatory architecture in combination with expression plasticity over a broad range of developmental temperatures. We found that genes with expression differences triggered by developmental temperature could be grouped according to their reaction norms. Classifying the reaction norms based on the magnitude of expression differences (SOC), direction of change, and curvature, we noted some important features of the underlying regulatory architecture:

1. The number of TFs was the most important determinant of the regulation of plasticity.
2. The number of TFs is negatively correlated with expression dynamics (SOC).
3. TFs and microRNAs have opposing effects on the regulation of plasticity.
4. Using a model containing all regulatory features analyzed (TFs, microRNAs, length of first intron, and UTR length) the direction of gene expression change can be predicted with 80% accuracy.

Our observation that less dynamic genes (smaller SOC) are regulated by more TFs contrasts the recently observed positive correlation between expression fluctuation and number of TF-binding sites (Yang et al. 2012). One possible explanation for this discrepancy is a difference in experimental design. Although our study focused on changes in gene expression, which were consistent across replicates, the meta-analysis of Yang et al. (2012) identified variation across different experiments. Furthermore, we analyzed the number of different TFs binding to a target gene rather than the number of TF-binding sites, which may include multiple binding sites for the same TF.

### Patterns of Plasticity Identify Genes with Similar Function

Until now, the number of studies linking gene expression plasticity to functional categories is still limited. Using only two temperatures Runcie et al. (2012) measured the plasticity of gene expression in sea urchins. Similar to our study, the authors distinguished increasing and decreasing gene expression and found that genes from some specific GO categories were overrepresented. Contrary to our study, which found ion channels and G-protein-coupled receptors to be enriched in the decreasing category, in sea urchin these genes were enriched in the increasing category. Similarly, ribosomal proteins and splicing factors were enriched in the increasing category in flies, whereas in the sea urchin these groups of genes were enriched in the decreasing category. Probably the key difference between the two studies is that the sea urchins were exposed to a hot temperature environment they never encountered before, whereas the temperature regime applied to flies was extreme, but certainly within the range that flies experience in their natural habitats. This difference is also reflected in the expression of the heat shock proteins *Hsp70* and *Hsp90*. Both were induced in the sea urchin, indicating an acute stress response, but in our experiment we detected no upregulation at these Hsps, not even at the most extreme temperatures. Hence, we conclude that the inconsistent response to the temperature treatments between the two species may be the outcome of the different stress levels.

### Stability of Gene Expression Differences Induced by Developmental Temperatures

The expression differences seen between developmental temperatures are a combination of expression differences that only depend on the temperature shortly before the analysis and those that are determined during development and remain stable when flies are shifted to a different temperature. A recent study in *Caenorhabditis remanei* analyzed expression plasticity for individuals exposed for 20 h to two different temperatures (Sikkink et al. 2014). Similar to the results of this study, the authors noticed an enrichment of biological processes related to metabolism and growth while ion transport and cellular communication were downregulated at the hot environment of 30 °C. Future studies specifically designed to provide insight on the stability of gene expression patterns due to developmental temperature are needed. The similarity of gene expression changes in these two species, however, suggests that many of the expression differences seen in our study may not be stably induced during development, but represent a plastic response that could vary during the lifetime of individuals. One interesting difference in gene expression patterns between the *C. remanei* study and our *D. melanogaster* analysis is that in *C. remanei Hsp70* and *Hsp90* were upregulated in the stressful hot environment, whereas no upregulation was seen in flies exposed to the elevated temperature throughout their entire development. This difference suggests that short-term stress response can be seen even at nonextreme temperatures (Sikkink et al. 2014), but not for lifetime exposure. Although this makes biological sense given the costs associated with *Hsp70* and *Hsp90* upregulation (Parsell and Lindquist 1993), it needs validation in the same organism with identical genotypes.

### Are Temperature-Induced Gene Expression Changes Adaptive?

The strong enrichment of functional categories among genes sharing a similar plasticity pattern may suggest that the plasticity in gene expression is a response to altered functional requirements in the different temperature environments. One plausible example for this is the upregulation of ion transporters in cold environments: The lower conductivity of ion channels at low temperature may require more ion channels for homeostasis. Alternatively, the change in gene expression may simply reflect a biochemical or physiological interaction with the environment. It is conceivable that this applies to the increased expression of metabolism-related genes at a higher temperature. Hence, it is possible that these expression changes are neutral or even deleterious. This question about the fitness consequences of temperature-induced gene expression changes could be addressed by experimental evolution asking how the plasticity in gene expression is being modulated as response to different temperature environments. Alternatively, populations evolved in habitats with different temperature regimes could be compared for their pattern of plasticity (Levine et al. 2011).

## Materials and Methods

### Fly Preparation and sequencing

We used F1 offspring of two *D. melanogaster* inbred strains, Oregon R and Samarkand, to avoid possible misexpression caused by inbreeding (Kristensen et al. 2005; Jensen et al. 2014). All flies were reared on standard cornmeal–molasse–yeast–agar medium and maintained in 12 h light/12 h dark conditions. The parental strains were inbred for seven generations by brother–sister mating to remove residual variation. Virgin females of either strain were collected and used for F1 crosses: O female × S male (cross F1_A_), and S female × O male (cross F1_B_). For each type of cross, approximately 240 mating pairs were set up in individual vials and randomly divided into three replicate groups of 80 vials. After 2 days of egg laying at 25 °C, these 80 vials per replicate were divided into four subsets of 20 vials that were moved to four different temperatures (13, 18, 23, and 29 °C). Virgin F1 females were collected after eclosion and aged 3 days at the respective temperature before shock-freezing in liquid nitrogen. For each group, approximately 30 females were homogenized in peqGOLD TriFast Reagent (Peqlab, Erlangen, Germany) using an Ultraturrax T10 (IKA-Werke, Staufen, Germany). Total RNA was extracted, quality-checked on agarose gels, and quantified using the Qubit RNA Assay Kit (Invitrogen, Carlsbad, CA). Paired-end Illumina mRNA libraries were generated from 5 µg total RNA: After DNase I treatment (Qiagen, Hilden, Germany), poly(A) transcripts were isolated using the NEBNext Poly(A) mRNA Magnetic Isolation Module (New England Biolabs, Ipswich, MA). Strand-specific paired-end libraries were prepared using the NEBNext Ultra Directional RNA Library Prep Kit and size-selected on AMPureXP beads (Beckman Coulter, CA) aiming for fragments between 380 and 500 bp. All 24 (four temperatures each three replicates for both parent-of-origin crosses) libraries were amplified with 12 polymerase chain reaction cycles using index primers from the NEBNext Multiplex Oligos for Illumina Kit (New England Biolabs) and sequenced on a HiSeq2000 using a 2 × 100 bp protocol. All sequences have been deposited in the NCBI Sequence Read Archive with the accession number SRP041395 (Chen et al. 2015).

### Reaction Norms of Gene Expression

Sequence reads were trimmed using the Mott algorithm implemented in PoPoolation (Kofler et al. 2011) and aligned to the *D. melanogaster* 5.49 assembly using GSNAP (Wu and Watanabe 2005). Only proper-paired reads with unique mapping position were used and only a fraction of mismatches less than 2% was allowed (-m 0.02 -s –split-output), which retrieved on average 77% of read pairs for each sample. We added gene models from the developmental transcriptome (Graveley et al. 2011) to the ones in Flybase in case they did not overlap with gene regions described by Flybase. This resulted in a total number of 18,764 gene models. Levels of gene expression were measured as paired-end read counts using the ReCOG software tool (https://code.google.com/p/recog/, last accessed May 25, 2015). We only counted read pairs that were mapped fully within the gene boundaries. Read pairs were considered ambiguous and not counted if mapped across gene boundaries or to the overlapping region of multiple genes. We defined genes as expressed if at least one read was mapped in each of the samples and at least one sample had ≥20 counts (McManus et al. 2010; Goncalves et al. 2012). Read counts of each gene were normalized by the total library size and RNA composition of each data set using a TMM (Robinson and Oshlack 2010). To account for RNA composition, a set of scaling factors for each library size were computed that minimized the log-fold changes between samples for most genes. We used the product of the total read number and the scaling factor as the effective library size for each replicate. For normalization, the effective library sizes were introduced into the GLM as an offset vector. We then applied a GLM method with negative-binomial distribution to account for the overdispersion of count data. Only genes expressed at all four temperatures were used to model reaction norms. Instead of pairwise comparisons, we treated the temperature as a continuous variable and calculated the trend of gene expression changes across all four temperatures. We modeled both linear and quadratic reaction norms on temperature using the equations

(1) $$e=E_{a}+g_{1}(t-T_{a})$$

and

(2) $$e=E_{m}+g_{2}{\left(t-T_{m}\right)}^{2}$$

(modified from: Gibert et al. 1998), where the expression vector (*e*) can be described by the slope (*g*_1_), temperature (*t*), midpoint temperature (*T_a_*), and the average response (*E_a_*) in linear equation (1); or by the quadratic coefficient (*g*_2_), optimal temperature (*T_m_*), and the expression at *T_m_* (*E_m_*) in quadratic equation (2). To determine the linear and quadratic reaction norms, we carried out model selection using likelihood ratio tests with a significance level ≤0.05. All *P* values were adjusted by correction (Benjamini and Hochberg 1995). We considered a gene as differentially expressed across temperatures only if a significant temperature coefficient (FDR ≤ 0.05) was observed as well as a max/min expression ratio over 1.25. Analyses were performed on the combined data set of F1 crosses from both directions as no imprinting effects could be detected (Chen et al. 2015).

Genes with significant expression difference were further assigned to four classes according to their direction of gene expression change along temperature:

**Table:** 

Class I	—increasing: g along temperature following either a linear curve or a quadratic curve.
Class II	—decreasing: Genes with the expression level decreasing along temperature following either a linear curve or a quadratic curve.
Class III	—U-shaped: Genes with the expression level following a quadratic curve with the parabola opening upward. To distinguish between increasing/decreasing quadratic curves, we further required the expression difference between 13 and 29 °C to be less than 80% of that between minimum and maximum expression levels among four temperatures.
Class IV	—bell-shaped: The same criterion was applied as for genes with U-shaped reaction norms but with the parabola opening downward.

### Linking the Regulatory Architecture with Plasticity Patterns

We determined three factors to evaluate the plasticity patterns in our experiment. We first quantified the dynamics of expression change across temperatures by calculating the SOC:

(3) $$\text{SOC}=\frac{\sum_{i=1,j=i+1}^{n-1}\left(\log_{2}\text{Exp}r_{\left(T_{j}\right)}-\log_{2}\text{Exp}r_{\left(T_{i}\right)}\right)}{\sum_{i=1,j=i+1}^{n-1}\text{abs}(\log_{2}\text{Exp}r_{\left(T_{j}\right)}-\log_{2}\text{Exp}r_{\left(T_{i}\right)})},$$

where log_2_-based fold-changes were calculated between two temperatures differing by no more than 6 °C. The second plasticity factor considered was whether the expression increased or decreased in expression intensity (direction) and the third plasticity factor distinguished between linear or quadratic reaction norms (curvature). We used 149 TFs and 148 microRNAs and their target genes from the Drosophila Interactions Database version 2013-07 (Murali et al. 2011). These databases only record experimentally verified interactions. We modeled the transacting regulatory architecture by summarizing the number of TF/microRNA-target interactions for each gene. Each TF/microRNA-target gene combination was counted once-thus multiple binding sites of the same TF/microRNA were treated as a single one. To model the *cis*-acting components of the regulatory architecture, we used the length of UTRs (5′-UTR and 3′-UTR) and the size of first intron in the longest transcript for each gene. We used the following regression model:

(4) $$\text{Plasticity}∼{\text{TFs+microRNAs + UTRS'+UTR3'+Intron}}^{\text{1st}}\text{+ ε,}$$

where plasticity indicates each of the following three plasticity components: SOC, the direction of expression change, and curvature, and *ε* denoted the error term. We used GLM to regress SOC (fourth root transformed) on the components of the regulatory architecture and assumed a Gaussian distribution for *ε*. The coefficients and *P* values were evaluated simultaneously for three plasticity components. We used the AIC to keep only those factors of the regulatory landscape that contributed significantly (i.e., the number of TFs and microRNAs). The effect size of each component was estimated using the reduction of the explained deviance (*R*^2^), which is the difference of the total explained deviance between a full model and a reduced model with only two plasticity factors and their interactions (e.g., *R*^2^_TFs_ = *R*^2^_(TFs+microRNAs)_ − *R*^2^_(microRNAs)_).

We performed linear discriminant analyses (LDA) to evaluate the contribution of the components of the regulatory architecture to the direction of expression change and curvature. We randomly selected 60% the loci from the total data set as a training data set. The remaining 40% of the genes were used to evaluate the classification accuracy based on the five components of the regulatory architecture. We followed the same strategies to also calculate the accuracy but using the random forest analysis implemented in the R package “randomForest” (Liaw and Wiener 2002), which unlike LDA assumes a nonparametric model and is not affected by the normality of the data.

We note that genes with a U- or bell-shaped reaction norm were not included in our analysis of the regulatory architecture as for these plasticity classes the change in gene expression was not a monotonous function of gene expression. Given that only a relatively small number of genes fell into these classes we felt that their inclusion would result in a more complex but less powerful model.

### GO Enrichment Analyses

We performed GO enrichment analyses on each plasticity class using the hypergeometric distribution-based algorithm implemented in program Gorilla (Eden et al. 2009). Genes in a given plasticity class were used as target data set to compare against the total data set of 9,995 genes expressed in all temperatures. For bell-shaped and U-shaped reaction norms we also performed ranked GO enrichment analyses based on the values of the temperature coefficient, which represents how fast the level of gene expression changes with temperature. Selection of significantly overrepresented GO categories was based on FDR *q* value ≤ 1 × 10^−^^5^ to account for multiple testing. Heat shock proteins were identified from the UniprotKB database and 19 genes were examined in our expression data.

## Supplementary Material

Supplementary data sets S1 and S2, table S1, and figure S1 are available at *Molecular Biology and Evolution* online (http://www.mbe.oxfordjournals.org/).

Supplementary Data
