# Supplementary material for: Temperature-Related Reaction Norms of Gene Expression: Regulatory Architecture and Functional Implications
Source: Mol Biol Evol. 2015 May 14;32(9):2393–402. doi: 10.1093/molbev/msv120 (PMC4540970; doi:10.1093/molbev/msv120)
Supplement: Supplementary Data [file supp_32_9_2393__index.html]

Temperature-Related Reaction Norms of Gene Expression: Regulatory Architecture and Functional Implications — Temperature-Related Reaction Norms of Gene Expression: Regulatory Architecture and Functional Implications — Supplementary Data 

# Temperature-Related Reaction Norms of Gene Expression: Regulatory Architecture and Functional Implications

## Supplementary Data

files

- Supplementary Data - xls file
- Supplementary Data - xls file
- Supplementary Data - pdf file
