# Supplementary material for: Temperature-Related Reaction Norms of Gene Expression: Regulatory Architecture and Functional Implications
Source: Mol Biol Evol. 2015 May 14;32(9):2393–402. doi: 10.1093/molbev/msv120 (PMC4540970; doi:10.1093/molbev/msv120)
Supplement: Supplementary Data [file supp_msv120_Chen_Suppl.pdf]

Supplementary

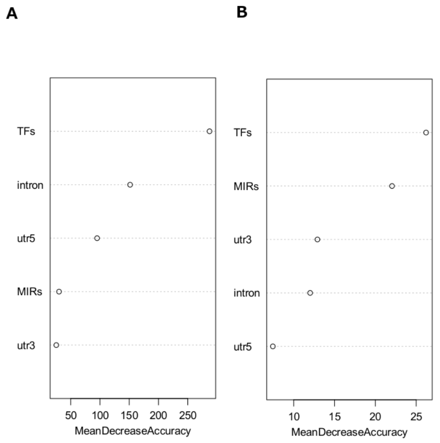

Figure S1 Dot chart of variable importance as measured by a random forest analyses (A) classification of direction (B) classification of curvature

Table S1. Mean number of read pairs mapped across three replicates are given for in $F1_A$ and $F1_B$ at four temperatures

	Temperature	13°C	18°C	23°C	29°C
**$F1_A$**	#raw reads	43,672,116	40,889,194	41,640,367	45,117,839
	#reads mapped	29,878,137	30,093,347	31,965,901	37,138,250
	#reads mapped to features	23,532,169	24,828,558	27,181,009	32,809,422
**$F1_B$**	#raw reads	42,796,478	42,474,298	42,725,914	43,042,101
	#reads mapped	29,879,783	31,433,611	33,216,419	35,892,684
	#reads mapped to features	23,971,573	25,739,283	28,357,561	32,057,227

Dataset S1 Reaction norm coefficients and classification of all expressed genes across temperatures

Dataset S2 Enriched GO categories for groups of genes classified based on expression curvatures or directions.
